# Knowledge and Perception of Diabetes and Available Services among Diabetic Patients in the State of Qatar

**DOI:** 10.5195/cajgh.2019.333

**Published:** 2019-01-24

**Authors:** Al-Anoud Al-Thani, Aiman Farghaly, Hammad Akram, ShamsEldin Khalifa, Benjamin Vinodson, Alma Loares, Abdul-Badi Abou-Samra

**Affiliations:** 1 Ministry of Public Health, State of Qatar; 2 Department of Medicine, Hamad Medical Corporation, State of Qatar

**Keywords:** Diabetes, Diabetes Knowledge, Diabetes Health Services, Diabetic Patient Survey, Qatar

## Abstract

**Introduction:**

Diabetes is a major public health concern in Qatar. This study examined diabetes knowledge and perception of available services for diabetes control among diabetic patients in Qatar.

**Methods:**

Data from 300 diabetic patients were collected through face-to-face interviews using a semi-structured questionnaire between February and May 2015 at Hamad Medical Corporation healthcare facilities in Qatar. Survey responses were represented as frequencies, and Chi-square tests were used to compare proportions across gender. A p-value of 0.05 was considered statistically significant.

**Results:**

31% of patients had Type 1 Diabetes (T1D) (females 36.6%, males 26.5%) and 54% had Type 2 Diabetes (T2D) (males 56.6%, females 50%). Knowledge about diabetes types did not differ by sex (P=0.16). 32.3% of patients were treated for diabetes-related complications including: high cholesterol (39.2%), vision problems (33.1%), hypertension (30.0%), and foot problems (25.1 %). Most patients were diagnosed at primary care clinics (41.7%). During visits, 78.3% of patients reported that they were fully advised about different diabetes tests. 57.0% of patients had ≥4 visits for diabetes checkups in the past 12 months. 66.7% of patients reported that they were confident or very confident in managing their diabetes as a result of their healthcare visits in the past year. The majority of patients reported receiving diabetes-related guidance from physicians (89.7%).

**Conclusions:**

Study participants had variable knowledge of diabetes, its complications and risk factors, and services available to diabetics. More comprehensive education and awareness about diabetes is recommended for both patients and family members. At the provider level, further improvement in patient counseling and promotion of available services can be beneficial.

## Introduction

Morbidity and mortality associated with chronic diseases are growing in Qatar. Diabetes is one of the major public health problems in Qatar, which requires attention of policy makers, clinicians, and public health officials. Risk factors associated with diabetes, such obesity and sedentary lifestyle, are common in Qatar.^1^, ^2^ As evident from a national population-based survey conducted in 2012, the prevalence of diabetes among Qatari nationals was 16.7%, which is significantly higher compared to 11.6% reported in a 2006 survey.^3^,^4^ Recent studies also indicate that the diabetes prevalence is associated with social and behavioral characteristics of the population in Qatar, including obesity, low educational status, marital status (ever or currently married), older age, and a family history of diabetes.^3^,^5^ The high burden of diabetes and related risk factors inspired the development and implementation of the Qatar National Health Strategy (NHS) aimed at preventing, monitoring, and educating patients and the general public about diabetes.

Diabetes was recognized as one of NHS’s high-priority diseases for preventive healthcare.^6^ An NHS report revealed that in 2015, chronic diseases such as heart disease, cancer, and diabetes, were responsible for 70% of the mortality in Qatar, with 9% of total mortality in Qatar attributed to diabetes.^6^ The NHS target is to reduce overall cause-specific mortality due to the preventable diseases to 5%, and preventable hospital admissions (that can be addressed in primary care setting, such as diabetes-associated health issues) to 15%.^6^ Through the National Diabetes Strategy (NDS), the diabetes initiative aimed to decrease the incidence and complications of diabetes by raising public awareness through adoption of multipronged health promotion approaches.^7^

In 2015, the Ministry of Public Health (MOPH) of Qatar, in collaboration with YouGov (an international market research organization), carried out a comprehensive research study examining the burden of diabetes, availability of services for diabetes management, public perception of diabetes, knowledge of diabetes by diabetic patients and the general population, and diabetes-related health system access in the State of Qatar. The aim of this collaborative project was to improve general public awareness and education on behavioral and lifestyle choices for diabetes prevention. Our present study describes the results from a diabetic patient-based survey and examines findings from patients who sought medical care at local hospitals and healthcare facilities in Qatar. We intend to present demographic data on diabetic patients, participant knowledge of diabetes-related complications and risk factors, and participant perception on diabetes-related services in the State of Qatar.

## Methods

This study was conducted at the major public hospitals and clinics of Hamad Medical Corporation (HMC) healthcare system, serving patients from all socioeconomic groups. HMC is the main public non-profit health care provider offering about 90% of acute services in the State of Qatar. 8–10 Quality and accessible healthcare can be accessed using a Health Card system at HMC, which provides subsidized and/or free health services to citizens and residents.^9^,^10^ For this study, participants were recruited from the adult and pediatric diabetes clinics, women’s clinics, renal centers, and podiatry clinics. Qatar residents (Qatari and Non-Qatari nationals) of both sexes who were 16 years or older with diabetes were included in the study. Individuals who were under 16 years of age, did not have diabetes, and non-residents of Qatar (e.g. visitors), were excluded. Patients were approached in the waiting areas or walk-in rooms of these healthcare facilities. Using a purposive sampling approach, the trained research personnel carried out the surveys during working hours of these health facilities until the study reached the target sample of 300 individuals. The 20–30 minute survey was carried out between February and May of 2015. A pre-tested and validated English and Arabic-translated semi-structured questionnaire was utilized to collect the data. Study participants were then categorized into Qataris, Arab expatriates, Asian expatriates, and Caucasian expatriates. No personal identifiers were collected for this survey. Sample selection was based on gender, age, and nationality representation as described elsewhere. 11 The age groups were corresponded to the approximate 2015 Qatar census proportion. 11

Survey questions were used to obtain patient data on the general demographic characteristics, knowledge about diabetes-related complications, risk factors and management, and degree of diabetes service level support received from health facilities, health providers, and other local diabetes support groups. Knowledge and perception of diabetes and diabetes-related factors were measured using a 5-point Likert scale question style (nothing, very little, some, enough, and a lot).

Ethical procedures were followed during the survey implementation and data handling procedures. Informed (verbal) consent was obtained before the survey. Patients were assured that the collected information would only be used for scientific purposes without sharing personal or identifiable information with third parties. Parental consent was obtained for respondents below 18 years of age. Supreme Council of Health, Doha, Qatar (now MOPH) provided ethical approval for this protocol.

Participant characteristics, including demographics, diabetes diagnosis and management, and diabetes support were reported using frequency and percentages. Chi-squared tests or Fisher exact test (where response frequency was less than 5) were performed to compare categorical variables by gender. Mann-Whitney test was used to compare duration of diabetes by gender. The threshold level of significance was set at p<0.05. Statistical analyses were performed using the SPSS Software version 22.0 (IBM Corporation, Chicago, IL, USA).

## Results

A slightly higher percentage of males (55.3%) participated in this study. The highest percentage of participants were in the age group of 35–54 years (41.0%), followed by 25–34 years (24.0%). Arab expatriates comprised 36.7% of the participants, followed by Asian expatriates (30.7%) and Qataris (29.3%). Most of the respondents were married with children (80%). Details about study participants are outlined in Table 1.

**Figure 1:**
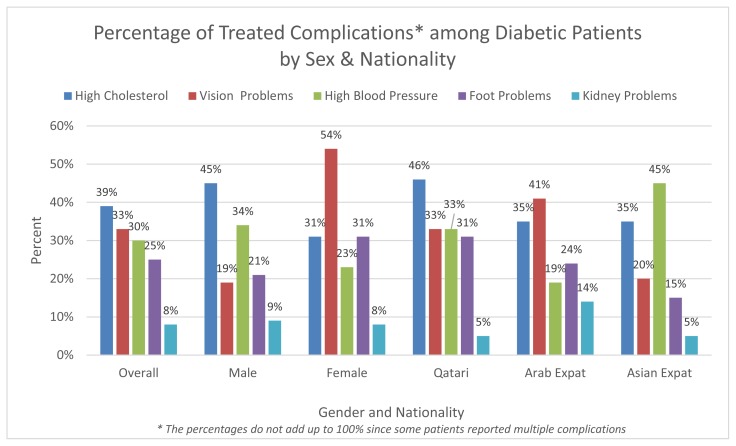
Percentage of treated complications among diabetic patients

Among overall sample, 31.0% of respondents reported having type 1 diabetes (T1D), 53.7% type 2 diabetes (T2D) and 15.3% did not specify the type (Table 2). Among men, 56.6% reported T2D and 26.5% T1D while among females 50.0% and 36.6% reported to have had T2D and T1D respectively (table 2). The proportion of respondents knowledgeable about their type of diabetes did not significantly differ by sex (P =0.16). About 32.3% of respondents who were treated for diabetes complications (single or multiple) reported having high cholesterol (39.2%), vision problems (33.1%), hypertension (30.0%), and foot problems (25.1%) (Figure 1). Most of the respondents were diagnosed with diabetes at primary care clinics (41.7%). 44.3% reported that they received sufficient diabetes-related information. About 78.3% of survey participants were fully advised regarding the different types of diabetes tests; a significantly higher proportion of females (82.1%) compared to males (75.3%) reported that they were fully advised (P=0.009).

More than half of patients (57.0%) had four or more visits for their disease-related checkups in the past 12 months. The majority of patients received diabetes-related guidance from physicians (89.7%) (Table 3). Most of the patients (66.7%) reported that they were confident or very confident in managing their diabetes themselves as a result of their diabetes follow-up visits during the past 12 months. Responses regarding number of patients undergoing diabetes related tests ranged from 46.7% (for foot examination) to BMI or body weight calculation (79.3%) (Table 3).

Moreover, 82.3 % of participants reported that doctors at the clinics would be able to provide them with diabetes-related support (Table 4). 64.3% of participants expressed their interest in talking with other diabetes patients. 15.7% contacted diabetes support or advocacy groups, 24.0% despite knowing advocacy groups did not contact them, and 60.3% of patients were not aware of diabetes support or advocacy groups.

It is important to note that more than half of the patients had no idea about the effect of drinking alcohol (58.0%) or smoking (53.3%) on diabetes. Furthermore, 47.7 % had no (33.0%) or very little knowledge (14.7%) while 52.3% had some (23.3%), enough (17.0%) and a lot (12.0%) knowledge about the adverse effects of taking diabetes medications (Table 5).

## Discussion

Diabetes is one of the top causes of death in the State of Qatar.^12^ Of the 300 patient-respondents, more than half were found to have T2D, with males having a slightly higher prevalence than females. A higher prevalence of T2D among males was also reported in other local and regional studies.^13^–^22^ Common identified complications were high blood cholesterol concentrations, hypertension, vision, and foot problems. Primary care clinics (42%) were identified as the primary place where patients were diagnosed with diabetes. The majority of respondents indicated that they have at least “some” or “enough” knowledge about most of the risk factors influencing diabetes (Table 5). Moreover, more than half indicated that they were informed “enough” or “a lot” about the importance of having regular visit with healthcare providers (65.3%).

Qatar’s diabetes registry for 2014–2016 showed that out of 2000 registered patients, about 95% had T2D, with the remaining having T1D, pre-diabetes and secondary diabetes respectively, indicating a high prevalence of T2D among diabetic patients.^23^ By 2050, both the prevalence and incidence of T2D are forecasted to increase by at least 43% and 147%, respectively in Qatar.^24^ The rise in T2D prevalence is projected to increase national diabetes related health expenditure from 20% currently to 32% by 2050.^24^

Our results showed a relatively higher than expected proportion of T1D (31.0%) among diabetic patients surveyed for Qatar. This is likely due to the fact that the survey was carried out at a tertiary hospital setting. Another possible explanation of the high percentage of T1D among participants could be the high prevalence of vitamin D deficiency among adults and children in Qatar reported in previous studies.^25^–^27^ These studies suggested a possible relationship between vitamin D deficiency and T1D; however, more studies are needed to explore this relationship in Qatar.^26^,^27^

Almost one-third of the patients surveyed were receiving treatment for diabetes-related complications. According to the National Health and Nutrition Examination Survey (NHANES), 1999–2004 (USA), the prevalence of diabetes complications among diabetics in the US were mainly due to kidney disease (27.8%), foot problems, (22.9%), and retinopathy (18.9%).^28^ In a Saudi review article investigating diabetes-related complications, the frequency of neuropathy and foot disease combined were alarmingly high at 82.0%, and the prevalence of retinopathy was 31%, and the prevalence of kidney disease requiring dialysis was between 30% to 45%.^29^,^30^ In comparison to NHANES data, only 8% of patients were receiving treatment for kidney issues, 25.1% for foot problems, and 33.1% for vision problems in our sample.^28^

We found that most of the patients received care at primary healthcare clinics and emergency rooms, and about half reported that they received adequate information about their diagnoses. Almost all the patients who received brochures during their visits were happy about them. Our survey findings provided valuable information because patient satisfaction in most healthcare services predicts the quality of care and management given by health providers.^31^

We found that most patients reported that they were confident in managing their disease after 12 months of checkups. In terms of patient-provider relationship, the majority of patients received guidance from their doctors and they were fairly able to work with healthcare professionals to set goals on the best ways to manage their diabetes. As expected, most patients cited doctors as their primary source of knowledge about their disease.^32^

The patient-provider relationship is important since it is evident that lack or insufficient communication between provider and patient with diabetes could lead to poor compliance.^33^, ^34^ Furthermore, the patients who less frequently receive information about diabetes are also less likely to manage their disease themselves.^35^ In our study, the participants understood the importance of relationships with healthcare providers, family members and other diabetics for support, and advocacy groups. In another study, the patient-provider collaboration, positive attitude, social support, and participation in group educational activities were considered as the essential components of effective diabetes self-management strategy.^36^

In general, a greater proportion of respondents had at least some understanding of their diagnosis and disease, such as the significance of their blood glucose levels, cholesterol levels, blood pressure, and disease management (maintaining weight, having regular check-ups, checking their feet, and other preventative behaviors). On the other hand, more than half had no or very little understanding of specific diabetes-related factors such as alcohol use, smoking, and medication side effects. Although our findings might not be comparable due to differences in the survey used, a study conducted in the UAE showed low levels of overall awareness on diabetes, while we found a higher degree of diabetes knowledge and awareness among our study participants.^32^

This study examined perception and knowledge of services, risk factors, symptoms and certain disease risk factors among diabetic patients. Certain diabetes-related knowledge areas e.g. smoking, alcohol use, and adverse effects of medicines, require further education and counseling. Patients were found to be generally satisfied regarding the level of information and support they received from the healthcare system. It would be beneficial to further expand diabetes-related programs in Qatar by taking innovative approaches, such as diabetes hotline numbers, using social media, and health campaigns and events to address disease-related concerns. This study was an initial attempt to understand the patient perspective on diabetes and services provided to them. The findings from this study will be beneficial in garnering support from key stakeholders and policymakers for diabetes programs in Qatar and will help in further strengthening ongoing projects targeting the country’s diabetes epidemic. Application of appropriate surveillance approaches including that can also be used for other conditions are essential in studying diabetes and diabetes-related factors, especially factors that have already been identified in the country.^2^,^5^,^37^–^45^

## Figures and Tables

**Table 1:** Demographic characteristics of the sample

Characteristics	n (%)
Age	
16–24 years	20 (6.7)
25–34 years	72 (24.0)
35–54 years	123 (41.0)
55–64 years	58 (19.3)
65 years and above	27 (9.0)
Gender	
Male	166 (55.3)
Female	134 (44.7)
Nationality group	
Qatari	88 (29.3)
Arab Expatriates	110 (36.7)
Asian	92 (30.7)
Caucasian	10 (3.3)
Marital status	
Single	29 (9.7)
Married with children	240 (80.0)
Married without children	18 (6.0)
Widowed/divorced	13 (4.3)
**Total**	**300**

**Table 2:** Sample characteristics by diabetes types, duration, and diagnosis associated factors

	Total, n (%)	Male, n (%)	Female, n (%)	P value
Diabetes type (A1)				
T1D	93 (31.0)	44 (26.5)	49 (36.6)	0.16
T2DM	161 (53.7)	94 (56.6)	67 (50.0)	
Others/unspecified	46 (15.3)	28 (16.9)	18 (13.4)	
Duration of diabetes (A2)				
Months [Median(IQR)]	60 (24, 144)	61 (24, 132)	60 (24, 156)	0.70*
Treated with complications (A1c)				
Yes	97 (32.3)	58 (34.9)	39 (29.1)	0.28
No	203 (67.7)	108 (65.1)	95 (70.9)	
Location of diagnosis (A3)				
Screening bus	6 (2.0)	3 (1.8)	3 (2.2)	<0.001†
Primary care	125 (41.7)	77 (46.4)	48 (35.8)	
Diabetes clinic	39 (13.0)	22 (13.3)	17 (12.7)	
Emergency room	51 (17.0)	31 (18.7)	20 (14.9)	
Women’s hospital	23 (7.7)	-	23 (17.2)	
Professional test following a self-test	38 (12.7)	22 (13.3)	16 (11.9)	
Other	18 (6.0)	11 (6.5)	7 (5.2)	
Level of information at first time of diagnosis (A5)				
Too little information	113 (37.7)	67 (40.4)	46 (34.3)	0.45
Received right amount of information	133 (44.3)	72 (43.4)	61 (45.5)	
Received too much information	32 (10.7)	14 (8.4)	18 (13.4)	
Don’t know/can’t remember	22 (7.3)	13 (7.8)	9 (6.7)	
Brochures on diabetes at time of diagnosis (A6)				
Yes	148 (49.3)	74 (44.6)	74 (55.2)	0.07
No	146 (48.7)	90 (54.2)	56 (41.8)	
Don’t know	6 (2.0)	2 (1.2)	4 (3.0)	
Satisfied with brochures (A7)				
Extremely dissatisfied	3 (2.1)	1 (1.4)	2 (2.7)	0.11
Dissatisfied	-	-	-	
Neither satisfied/dissatisfied	11 (7.4)	6 (8.1)	5 (6.8)	
Satisfied	94 (63.5)	53 (71.6)	41 (55.4)	
Extremely satisfied	40 (27.0)	14 (18.9)	26 (35.1)	
Fully advised on all of diabetes tests (A9)				
Yes	235 (78.3)	125 (75.3)	110 (82.1)	0.009*
No	46 (15.3)	34 (20.5)	12 (9.0)	
Don’t know/can’t remember	19 (6.3)	7 (4.2)	12 (9.0)	
**Total**	**300**	**166**	**134**	

* Mann Whitney test;

† Chi-squared test

**Table 3:** Follow up visits and treatment planning

	Total, n (%)	Male, n (%)	Female, n (%)	P value*
**Diabetes follow up visits in the past 12 months**				
Once	26 (8.7)	16 (9.6)	10 (7.5)	0.79
Twice	39 (13.0)	20 (12.1)	19 (14.2)	
Three times	42 (14.0)	23 (13.9)	19 (14.2)	
Four times or more	171 (57.0)	97 (58.4)	74 (55.2)	
**Received advice and guidance in relation to diabetes**				
Physician	269 (89.7)	153 (92.2)	116 (86.6)	
Nurse	5 (1.7)	1 (0.60)	4 (2.9)	
Support groups	2 (0.70)	1 (0.60)	1 (0.90)	
Friends	2 (0.70)	2 (1.2)	-	
Family	9 (3.0)	2 (1.2)	7 (5.2)	
Dietician	6 (2.0)	3 (1.8)	3 (2.2)	
Personal trainer	2 (0.70)	2 (1.2)	-	
Others	3 (1.0)	-	3 (2.2)	
Nobody	2 (0.70)	2 (1.2)	-	
**Value of advice and support provided by nurse**				
Yes	208 (69.3)	106 (63.9)	102 (76.1)	0.01
No	57 (19.0)	33 (19.9)	24 (17.9)	
**Worked with the health professionals to set goals about the best way to manage diabetes**				
Yes, completely each time I visit them	67 (22.3)	32 (19.3)	35 (26.1)	0.09
Yes, to some extent-sometimes at visit	101 (33.7)	67 (40.4)	34 (25.4)	
No, but I would have liked to	77 (25.7)	38 (22.9)	39 (29.1)	
**Received advice to change diet that could help manage diabetes**				
Yes, definitely	103 (34.3)	57 (34.3)	46 (34.3)	0.85
Yes, to some extent but not enough	82 (27.3)	47 (28.3)	35 (26.1)	
No, but I would have liked help/advice	56 (18.7)	33 (19.9)	23 (17.2)	
**Received advice on physical activity from clinic staff**				
Yes, definitely	128 (42.7)	79 (47.6)	49 (36.6)	0.001
Yes, to some extent but not enough	63 (21.0)	38 (22.9)	25 (18.7)	
No, but I would have liked help/advice	42 (14.0)	27 (16.3)	15 (11.2)	
**Thinking back to your last visit, were you given a copy of your diabetes plan?**				
Yes	172 (57.3)	103 (62.1)	69 (51.5)	0.11
No	119 (39.7)	60 (36.1)	59 (44.0)	
**Diabetes plan includes any of the following:**				
Your next appointment time and place	164 (95.3)	100 (97.1)	64 (92.8)	
Name of contact person	71 (41.3)	40 (38.8)	31 (44.9)	
Info. on managing diabetes between appointment	56 (32.6)	33 (32.0)	23 (33.3)	
Personal goal and targets about diabetes	43 (25.0)	31 (30.1)	12 (17.4)	
Advice on your diet and what foods to eat	67 (38.9)	41 (39.8)	26 (37.7)	
Advice on physical activity	51 (29.6)	34 (33.0)	17 (24.6)	
Your results of the diabetes tests	92 (53.5)	50 (48.5)	42 (60.9)	
A plan for medicines & lifestyle	91 (52.9)	58 (56.3)	33 (47.8)	
Your health information & diabetes status	45 (26.7)	18 (17.5)	27 (39.1)	
**Confidence in managing diabetes due to the health check-up in past 12 months**				
Very confident	66 (22.0)	42 (23.5)	27 (20.2)	0.006
Confident	134 (44.7)	84 (50.6)	50 (37.3)	
Not sure	77 (25.7)	35 (21.1)	42 (31.3)	
Fairly confident	20 (6.7)	5 (3.0)	15 (11.2)	
**Sometimes, a health professional will use complex medical terms and words that are not always understood by patients. Has this ever happened to you?**				
Yes	47 (15.7)	16 (9.6)	31 (23.1)	0.002
No	247 (82.3)	148 (89.2)	99 (73.9)	
Don’t know/can’t remember	6 (2.0)	2 (1.2)	4 (3.0)	
**In the last 12 months, have you undergone any of the following diabetes related tests? (Yes/No)**				
Weight/Body Mass Index (BMI)	238 (79.3)	128 (77.1)	110 (82.1)	
Blood Pressure	234 (78.0)	135 (81.3)	99 (73.9)	
Cholesterol	208 (69.3)	129 (77.7)	79 (59.0)	
HbA1c (long term blood glucose test)	204 (68.0)	128 (77.1)	76 (56.7)	
Blood test for kidney function	158 (52.7)	92 (55.4)	66 (49.3)	
Urine test	220 (73.3)	126 (75.9)	94 (70.1)	
Foot examination	140 (46.7)	71 (42.8)	69 (51.5)	
Eye screening	161 (53.7)	79 (47.6)	82 (61.2)	
Don’t know/can’t remember	8 (2.7)	3 (1.8)	5 (3.7)	
**Were the results of these tests fully explained to you? (B14)**				
Yes, and I understood them clearly	181 (61.9)	87 (53.4)	94 (72.9)	0.007
Yes, But I did not really understand them	26 (8.9)	18 (11.0)	8 (6.2)	
No, I didn’t need them to be	41 (14.2)	30 (18.4)	11 (8.5)	
No, not at all	22 (7.5)	12 (7.4)	10 (7.8)	
Don’t know/can’t remember	22 (7.5)	16 (9.8)	6 (4.7)	

* Chi-Square test

**Table 4:** Local or Social Support on Diabetes in Qatar

	Total, n (%)	Male, n (%)	Female, n (%)	P value*
**Do you have a contact number for addressing concern about the diabetes**				
Yes	76 (25.3)	36 (21.7)	40 (29.9)	0.09
No	223 (74.4)	130 (78.3)	93 (69.4)	
Don’t know	1 (0.30)	0 (0.0)	1 (0.70)	
**Which of the following do you feel you would be able to receive support from in relation to your diabetes?**†				
Doctor at local clinic	247 (82.3)	137 (82.5)	110 (82.1)	
Nurse at local clinic	98 (32.7)	48 (28.9)	50 (37.3)	
Specialist consultant at hospital	101 (33.7)	61 (36.7)	40 (29.8)	
Specialist nurse at hospital	30 (10.0)	19 (11.4)	11 (8.2)	
Counselor	31 (10.3)	13 (7.8)	18 (13.4)	
Telephone helpline	22 (7.3)	9 (5.4)	13 (9.7)	
Support group	18 (6.0)	11 (6.6)	7 (5.2)	
Coordinator	15 (5.0)	9 (5.4)	6 (4.5)	
Other people with diabetes (other than a support group)	53 (17.7)	17 (10.2)	36 (26.9)	
Family friend	111 (37.0)	44 (26.5)	67 (50.0)	
Other	7 (2.3)	4 (2.4)	3 (2.2)	
None of these	5 (1.7)	4 (2.4)	1 (0.75)	
**Since you have been diagnosed with diabetes, would you want to talk to other people who also have diabetes?**				
Yes	193 (64.3)	110 (66.3)	83 (61.9)	0.43
No	107 (35.7)	56 (33.7)	51 (38.1)	
**Have you been able to meet and talk to other people with diabetes?**				
Yes	173 (57.7)	91 (54.8)	82 (61.2)	0.26
No	127 (42.3)	75 (45.2)	52 (38.8)	
**Are you aware of any local or national diabetes support / patient advocacy groups?**				
Yes, and I have contacted them	47 (15.7)	26 (15.7)	21 (15.7)	0.72
Yes, but I have not contacted them	72 (24.0)	37 (22.3)	35 (26.1)	
No	181 (60.3)	103 (62.0)	78 (58.2)	
**Would you be willing to participate in any future studies on diabetes in Qatar?**				
Yes	169 (56.3)	98 (59.0)	71 (53.0)	0.29
No	131 (43.7)	68 (41.0)	63 (47.0)	

* Chi Square test;

† Multiple responses

**Table 5:** Likert Scale Responses “To what extent do you understand the following factors in relation to your diabetes?”

	Nothing n (%)	Very little n (%)	Some n (%)	Enough n (%)	A lot n (%)
The effects of being ill: ex. Having flu	89 (29.7)	50 (16.7)	89 (29.7)	50 (16.7)	22 (7.2)
Maintaining weight	51 (17)	49 (16.3)	76 (25.3)	854 (28.3)	39 (13.1)
Blood glucose drops too low	17 (5.7)	65 (21.7)	95 (31.7)	75 (25.0)	48 (16.0)
Regular check-ups with doctor/nurse	12 (4.0)	26 (8.7)	66 (22.0)	111 (37.0)	85 (28.3)
Cholesterol levels	60 (20.0)	52 (17.3)	81 (27.0)	84 (28.0)	23 (7.7)
Blood pressure	59 (19.7)	53 (17.7)	89 (29.7)	74 (24.7)	25 (8.3)
Checking and looking after eyes	54 (18.0)	38 (12.7)	86 (28.7)	58 (19.3)	64 (21.3)
Checking and looking after feet	64 (21.3)	56 (18.7)	79 (26.3)	66 (22.0)	35 (11.7)
Drinking alcohol	174 (58.0)	18 (6.0)	29 (9.7)	34 (11.3)	45 (15.0)
Smoking	160 (53.3)	12 (4.0)	53 (17.7)	33 (11.0)	42 (14.0)
Stress	31 (10.3)	68 (22.7)	108 (36.0)	56 (18.7)	37 (12.3)
Tiredness	28 (9.3)	58 (19.3)	110 (36.7)	71 (23.7)	33 (11.0)
Adverse effects taking medication	99 (33.0)	44 (14.7)	70 (23.3)	51 (17.0)	36 (12.0)
Severe complications left untreated	24 (8.0)	46 (15.3)	93 (31.1)	67 (22.3)	70 (23.3)
